# Hypoxia-induced ALDH3A1 promotes the proliferation of non-small-cell lung cancer by regulating energy metabolism reprogramming

**DOI:** 10.1038/s41419-023-06142-y

**Published:** 2023-09-20

**Authors:** Yang Chen, Hongfei Yan, Lirong Yan, Ximing Wang, Xiaofang Che, Kezuo Hou, Yi Yang, Xuena Li, Yaming Li, Ye Zhang, Xuejun Hu

**Affiliations:** 1 Department of Respiratory and Infectious Disease of Geriatrics, The First Hospital of China Medical University, 110001 Shenyang, China; 2 Department of Medical Oncology, Chongqing University Cancer Hospital, Chongqing, China; 3 Department of Medical Oncology, The First Hospital of China Medical University, 110001 Shenyang, China; 4 Key laboratory of anticancer Drugs and Biotherapy of Liaoning Province, The First Hospital of China Medical University, 110001 Shenyang, China; 5 The First Laboratory of Cancer Institute, The First Hospital of China Medical University, NO.155, North Nanjing Street, Heping District, 110001 Shenyang, China; 6 Liaoning Province Clinical Research Center for Cancer, 110001 Shenyang, China; 7 Laboratory Animal Center, China Medical University, 110001 Shenyang, China; 8 Department of Nuclear Medicine, The First Hospital of China Medical University, 110001 Shenyang, China

**Keywords:** Cancer metabolism, Non-small-cell lung cancer

## Abstract

Aldehyde dehydrogenase 3A1 (ALDH3A1) is an NAD^+^-dependent enzyme that is closely related to tumor development. However, its role in non-small-cell lung cancer (NSCLC) has not been elucidated. This study aimed to clarify the mechanism of ALDH3A1 and identify potential therapeutic targets for NSCLC. Here, for the first time, we found that ALDH3A1 expression could be induced by a hypoxic environment in NSCLC. ALDH3A1 was highly expressed in NSCLC tissue, especially in some late-stage patients, and was associated with a poor prognosis. In mechanistic terms, ALDH3A1 enhances glycolysis and suppresses oxidative phosphorylation (OXPHOS) to promote cell proliferation by activating the HIF-1α/LDHA pathway in NSCLC. In addition, the results showed that ALDH3A1 was a target of β-elemene. ALDH3A1 can be downregulated by β-elemene to inhibit glycolysis and enhance OXPHOS, thus suppressing NSCLC proliferation in vitro and in vivo. In conclusion, hypoxia-induced ALDH3A1 is related to the energy metabolic status of tumors and the efficacy of β-elemene, providing a new theoretical basis for better clinical applications in NSCLC.

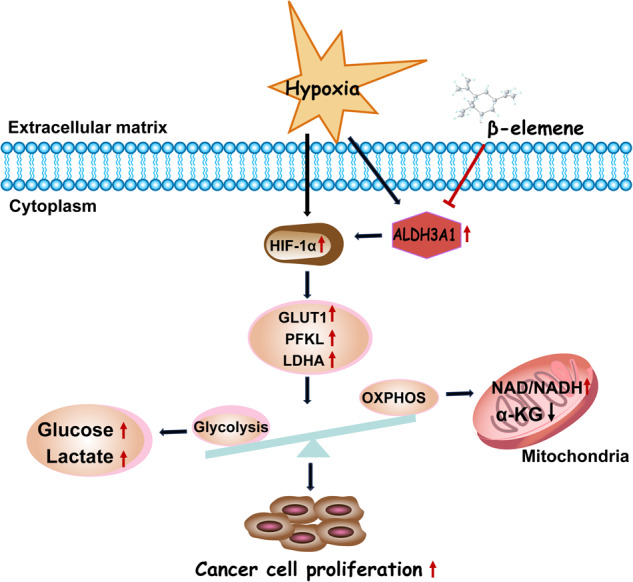

## Introduction

Hypoxic tumor microenvironments induce the glycolytic phenotype in many cancer cells [1]. While this hypoxic-induced increase in glycolytic flux is beneficial for promoting cell growth during hypoxic injury and is therefore adaptive in nature, the mechanisms surrounding this increased glycolytic flux can also be manipulated by cancer cells to promote their growth, even in the presence of adequate oxygen levels, a property of tumor cells known as the “Warburg effect” [2, 3]. The “Warburg effect” aims to meet the need of tumor cells for unlimited proliferation, which is characterized by active glycolysis, the accumulation of lactic acid, and a weakened oxidative phosphorylation process of mitochondria. These changes in energy metabolism are also called the energy metabolism reprogramming of tumors. Numerous studies have shown that the reprogramming of the energy metabolism of tumor cells can affect disease progression and efficacy [4–7]; therefore, new therapeutic strategies based on the reprogramming of energy metabolism in tumor cells have attracted significant attention.

Aldehyde dehydrogenase 3A1 (ALDH3A1) is an NAD^+^-dependent enzyme that oxidizes various endogenous and exogenous aldehydes to carboxylic acids [8]. Studies have demonstrated that ALDH3A1 expression is closely related to changes in the biological behavior of tumor cells, such as epithelial mesenchymal transition (EMT), metastasis, and cancer stem cell expansion, impairing immune surveillance [9–11]. In addition, one study indicated that the knockdown of ALDH3A significantly reduced ATP production and induced apoptosis in gastric cancer [12]. Exosomes carrying ALDH3A1 from irradiated lung cancer cells contribute to the motility of recipient cells by accelerating glycolysis [13]. A bioinformatics analysis showed that the glycolytic and gluconeogenic metabolic pathway mediated by ALDH3A1 was associated with p53 mutants and prognosis in lung adenocarcinoma [14]. Another study showed that the increased expression of ALDH3A1 was associated with drug resistance in NSCLC [15]. The exploration of ALDH3A1’s pathogenic mechanism in NSCLC has contributed to the discovery of effective therapeutic drugs targeting ALDH3A1 and combatting drug resistance, providing new clues for the identification of new precision therapeutic targets.

β-elemene is a monomeric antitumor compound extracted from turmeric. The previous studies of our team found that β‐elemene enhances the sensitivity of tumor cells to chemotherapeutic agents and has the potential to be a novel drug for multidrug-resistant cells, effectively preventing the proliferation, apoptosis, and metastasis of cancer [16–19]. Another study suggested that β-elemene could also inhibit breast cancer metastasis by inhibiting the translocation of pyruvate kinase M2 (a key glycolytic enzyme) to the nucleus [20]. However, the exact target of β-elemene is still undefined, leading to limitations on drug discovery and development.

In this study, we identified that ALDH3A1 is consistently upregulated and a biomarker of a poor prognosis in NSCLC. Interestingly, environmental hypoxia induced ALDH3A1 expression and ALDH3A1 mediated hypoxia-induced functions. Furthermore, ALDH3A1 induced energy metabolism reprogramming, promoted tumor growth, and was identified, for the first time, as a target of β-elemene in vivo and in vitro. In conclusion, this study suggests that hypoxia-induced ALDH3A1 is associated with the energy metabolic status of tumors and the efficacy of β-elemene, providing a new theoretical basis for better clinical applications.

## Materials and methods

### Antibodies and reagents

β-Elemene (molecular formula C15H24), whose molecular weight is 204.35, was provided by Jingang Pharmaceuticals (#081152, Dalian, China). The antibody of HIF-1α (#3716 S), LDHA (2012S), was obtained from Cell Signalling Technology (Danvers, MA, USA). The antibody GLUT1 (2646S) was purchased from NOVUS (USA), and the antibody PFKL (55028-1-AP) was purchased from Proteintech. Anti-PDK1 (ab226963) and Anti-PHD1 (ab113077) were purchased from Abcam. Anti-Ki67 antibody was purchased from Fuzhou Maixin Biological Technology (Fujian, China). ALDH3A1 (sc-137168), Cyclin B1 (166757), Cyclin D1 (8396), Cyclin E (sc-377100), and ACTIN (sc-47778) were purchased from Santa Cruz Biotechnology (Santa Cruz, CA, USA). 2-Deoxy-D-glucose (2-DG) was purchased from MedChemExpress (HY-13966, USA). The Cell Counting Kit-8 (CCK-8) was purchased from APExBIO (Catalog No. K1018, USA).

### Bioinformatics analysis

GSE18842 and GSE30979 were collected from Gene Expression Omnibus (GEO) (https://www.ncbi.nlm.nih.gov/gds/). The Limma R package was used to detect differentially expressed ALDH3A1. The correlation of ALDH3A1 with the overall survival of NSCLC cases was analyzed using the R package survminer. Lung adenocarcinoma data were downloaded from The Cancer Genome Atlas (TCGA, https://www.cancer.gov/tcga), which was used to perform Gene Set Enrichment Analysis (GSEA). The molecular docking was performed using PubChem (https://pubchem.ncbi.nlm.nih.gov/), the PDB (https://www.rcsb.org/) online website, and Schrodinger Suites software.

### Immunohistochemistry and immunofluorescence staining assay

Tumor histopathology specimens from 100 patients with lung adenocarcinoma were obtained from surgical specimens from the Department of Pathology of the Second Hospital of China Medical University from 2010 to 2013 and were approved by the Ethics Committee of China Medical University for use in the study (CMU2021037). The tumor sample preparation methods, as well as the staining intensity and staining area calculation methods, are described in our previous study [21]. The staining was evaluated by scanning the entire tissue specimen under low magnification (×10) and confirmed under high magnification (×20 and ×40). The protein expression was visualized and classified based on the percentage of positive cells and the intensity of staining. From each section, five visual fields were randomly selected. The degree of protein expression was based on the percentage of positive cells and the intensity of staining. Staining intensity was scored as 0 (no staining), 1 (low staining), 2 (intermediate staining), and 3 (high staining). For the staining area, ≤5%, 5–25%, 26–50, 51–75% and >75% were recorded as 0, 1, 2, 3 and 4 points, respectively. Histological score = staining intensity × staining area. A score 0 was classified as negative (−), 1–4 points as weakly positive (+) and 6–12 points as a strong positive (++). Final scores were assigned by three independent pathologists.

A total of 100 samples of NSCLC and adjacent normal tissues were acquired from patients who underwent surgical resection at the second hospital of China Medical University. Clinical information was collected. The participants were informed about the study’s ethical guidelines through written consent.

The immunofluorescence staining references are provided in our previous study [22].

### Cell culture

The NSCLC cell lines NCI-H1299 and A549 were obtained from the Chinese Academy of Sciences (Shanghai, China). All the cells were cultured in RPMI-1640 medium containing 10% heat-inactivated FBS at 37 °C in an atmosphere of 95% air and 5% CO_2_. The hypoxic conditions consisted of 1% O_2_, 5% CO_2_, and 94% N_2_.

### MTT assays

A total of 20 µl of the MTT reagent (5 mg/L) per well was added and incubated for another 3 h. The supernatant was removed, and 200 µl of dimethylsulfoxide (DMSO) was added. The absorbance (A) was measured at 570 nm.

### CCK-8 assays

After 24 h of transient transfection, the cells were digested, centrifuged, and counted. A cell suspension was prepared (each group had 3 secondary wells with 3000 cells per well), and 96-well plates were planted. We added 100 μl of cell suspension to each well and incubated the plates in the incubator for 3 h. After cell adhesion, 2-DG (4 mM) was added, and 10 μl of CCK-8 solution was rapidly added to each well at 0 h, 24 h, 48 h, and 72 h, respectively. After incubation for 2 h, the optical density (OD) at 450 nm was detected.

### Clone formation assay

NSCLC cells were plated at a rate of 300 or 4000 cells (NCI-H1299, A549) per well into 12-well plates in a medium containing 10% FBS. The cells were incubated at 37 °C in 5% CO_2_ overnight. After 10 days or 3 days, the number of colonies was counted via light microscopy after the cells were stained with Wright–Giemsa.

### EDU assay

Cells were deposited at a density of 3000 cells/well into 24-well plates. Referring to our previous method using an EDU kit (Ribbo Guangzhou, China), 3–5 representative sites of each sample were randomly counted and analyzed under a microscope (Olympus, Tokyo, Japan).

### Flow cytometry assay

NCI-H1299 and A549 cells were harvested and fixed with 70% (v/v) cold ethanol at 4 °C overnight. After 30 min incubation with 100 μg/ml of RNase A and 10 μg/ml of PI staining solution in the dark, the cells were analyzed using a FACScan flow cytometer (Becton Dickinson, USA). Cell Quest software (USA) and ModFit software were also used.

### Metabolite analysis

In our in vitro experiment, a glucose assay kit (BioVision) was used to check intracellular glucose uptake. The lactate level was quantified with a lactate assay kit (BioVision). The α-KG level and ratios of NAD^+^/NADH and NADP^+^/NADPH in the cell lysates were determined using BioVision for α-KG, AAT Bioques for NADP^+^/NADPH, and AAT Bioques for NAD^+^/NADH Ratio, respectively. All operations were performed according to the manufacturer’s instructions. In our in vivo assays, we applied MS-LC with mice tumor tissues. First, 200 μl of pre-cooled ultrapure water was added to 60 mg of each mouse tumor tissue for homogenization, and then 800 μl of pre-cooled methanol/acetonitrile (1:1, v/v) was added. The samples were separated using an Agilent 1290 Infinity LC ultra-performance liquid chromatography system. Mass spectrometry in the negative ion mode was performed using a 5500 QTRAP mass spectrometer (AB SCIEX). Finally, the peak areas and retention times were extracted using Multi quant software, followed by quality control and the identification of metabolites.

### RNA and lentivirus interference

For the interference of ALDH3A1, ViewSolidBiotech (Beijing, China) designed and prepared two siRNA sequences (Si1: 5′-GGAACUCAGUGGUCCUCAATT-3′ and Si2: 5′-UUGAGGACCACUGAGUUCCTT-3′) and a negative control siRNA (siNC). Lipofectamine 2000 (Invitrogen, Carlsbad, CA, USA) was used to transfect siRNA into cells according to the manufacturer’s protocol. Obio Technology Corp., Ltd (Shanghai, China) designed and undertook the lentivirus construction of ALDH3A1 knockdown cells with the GFP label. Polybrene was applied to transfect the A549 cell line with the sh-ALDH3A1 lentivirus. Control cells were transfected with an empty vector carrying GFP.

### RNA isolation and qRT-PCR

Trizol reagent (Invitrogen, Carlsbad, CA, USA) was used to isolate the total RNA of the A549, H1299 cells, and mice tissues. The total RNA concentration was quantified by measuring the absorbance at the wavelength of 260 nm. All reagents for reverse transcription (RT) were obtained from TaKaRa (Shiga, Japan). The PrimeScript™ RT Reagent Kit (Takara, Japan) was used for mRNA reverse transcription. Quantitative real-time PCR was performed using SYBR Premix Ex TaqII (TaKaRa) and measured with Applied Biosystems^®^ 7500 Real-Time PCR Systems (Thermo Fisher Scientific, Waltham, MA, USA). The internal control used was 18 S. The 2^−ΔΔCt^ method was employed to determine the fold change in the RNA expression of one sample compared to the calibration sample. The primer details are listed in Supplementary Table S1.

### Xenograft experiments

Four-week-old female BALB/c nude mice were purchased from Beijing Vital River Laboratory Animal Technology Co., Ltd (Beijing, China). Animals were raised in a pathogen-free environment in the Animal Laboratory of China Medical University and have obtained ethical approval from the Institutional Review Committee of China Medical University (ethical batch number: 2018127) for all in vivo experiments. The patient agrees to participate in this project and has also obtained ethical approval from the First Affiliated Hospital of China Medical University (ethical batch number: [2019] 2019-57-2). According to the Guidance for Institutional Animal Care and Use Committee of China Medical University, we euthanized the mice via CO_2_ inhalation when the tumor diameters reached 15 mm. A549 cells (3 × 10^6^) were suspended in 150 μl phosphate-buffered saline (PBS) and then subcutaneously (s.c.) injected into the right scapular region of the mice, while A549 cells (3 × 10^6^) expressing the empty vector carrying GFP (Sh-NC) or GFP-labeled ALDH3A1 knockdown lentivirus (Sh-ALDH3A1) were s.c. injected into the dorsal flanks of the mice. We applied a bilateral subcutaneous tumor formation model, so a total of 8 mice were selected. The mice were randomly divided into two groups when the size of the tumors reached approximately 100 mm^3^. One group orally received 0.9% saline as a control, while the other group received β-elemene 50 mg/kg via oral gavage every other day for 2 weeks. Measuring the tumor with a caliper each week, the body mass was also measured. The formula V = (width^2^ × length)/2 was applied to calculate the tumor volumes. Tumor volume measurements and mouse observers and group handlers were different experimenters.

### Micro-PET scan

The volume of ^18^FDG was a maximum of 0.1 ml with 11.1 MBq (300 μCi). The activity and measurement time were then recorded. Then, we injected ^18^FDG into the anesthetized tail vein and waited for 30 min. We transferred the model mice to a scanning bed and made the necessary adjustments. The posture of the mice ensured the stretching of their limbs, as configured for micro-PET (Metis-6) scanning, and Metis Console software was applied for the subsequent scanning and analysis.

### Western blot assay

Total protein was extracted for Western blotting according to our previous studies [18]. The proteins were detected with enhanced chemiluminescence reagent and visualized with Microchemi 4.2 (DNR Bio-Imaging Systems, Jerusalem, Israel).

### Seahorse XFp assay

Macrophages were seeded at 2 × 10^4^ cells/well in 96-well plates for 3 to 4 h to allow adherence to the plate. Then, they were treated with or without β-elemene overnight, the cells were changed to unbuffered assay media, and incubated in a non-CO_2_ incubator at 37 °C for 1 h. Oxygen consumption rates (OCR) and Extracellular Acidification rates (ECAR) were measured using an XF96 extracellular flux analyzer (Seahorse Bioscience) after the sequential addition of oligomycin or Glucose diluted in Seahorse XFp Base media, FCCP or Oligomycin diluted in Seahorse XFp Base media, and antimycin/rotenone or 2-deoxy-glucose (2-DG) diluted in Seahorse XFp Base media.

### Statistical analysis

The data were collected in three separate experiments and expressed as the means ± standard deviation (SD). Student’s two-tailed *t*-test methods were applied to calculate differences between groups. All analyses were undertaken using Rstudio or GraphPad Prism 8 software (*P*-value <0.05 was considered statistically significant).

## Results

### ALDH3A1 expression is upregulated and induced by hypoxia in NSCLC

**Fig. 1:**
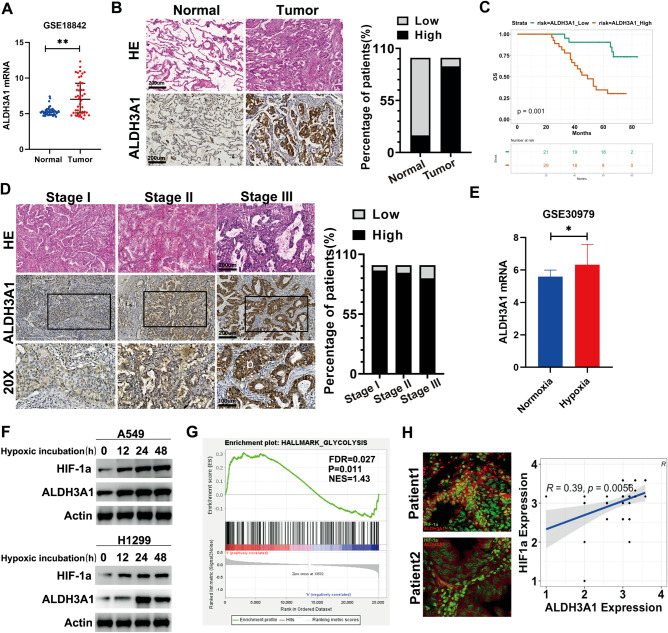
ALDH3A1 expression characteristics in NSCLC. **A** GSE18842 showed that ALDH3A1 was upregulated in NSCLC. **B** The expression of ALDH3A1 in normal and tumor tissues. **C** The Kaplan–Meier plot indicated that ALDH3A1 was associated with a poor prognosis of NSCLC. **D** Expression of ALDH3A1 in patients with different pathological stages. **E** GSE30979 showed that ALDH3A1 was upregulated in hypoxic patients with NSCLC. **F** ALDH3A1 and hypoxia-inducible factor-1α (HIF-1α) were significantly increased with hypoxia at 0, 12, 24, and 48. **G** GSEA enrichment analysis of ALDH3A1 in NSCLC with the TCGA dataset. **H** Immunofluorescence staining and correlation analysis of tissue samples. *P < 0.05, **P < 0.01, ***P < 0.001.

In the bioinformatics analysis using the GSE18842 dataset, we found that ALDH3A1 was highly expressed in NSCLC compared with the normal tissues (Fig. 1A). Our immunohistochemical staining indicated that ALDH3A1 showed a dramatically higher expression in the tumor tissues than in the normal tissues (Fig. 1B). Furthermore, ALDH3A1 expression was associated with a poor prognosis of NSCLC in the Kaplan–Meier plot (Fig. 1C). The percentage of patients with a high expression of ALDH3A1 was greater in stage III than in stage II and stage I in NSCLC (Fig. 1D).

The bioinformatics analysis using the GSE30979 dataset showed that compared with normoxic NSCLC patients, ALDH3A1 was upregulated in hypoxic NSCLC patients (Fig. 1E). To identify whether ALDH3A1 expression was induced by hypoxia in NSCLC, A549 and H1299 cells were cultured under both normoxic and hypoxic conditions. As a result, the expression of ALDH3A1 and hypoxia-inducible factor-1α (HIF-1α) were significantly boosted in a hypoxia time-dependent manner (Fig. 1F). GSEA indicated that ALDH3A1 was positively associated with glycolysis (Fig. 1G) and cell cycle checkpoints (Supplementary Fig. S1B) and negatively related to biological oxidations (Supplementary Fig. S1C). Furthermore, we applied immunofluorescence staining to identify the co-expression of ALDH3A1 and HIF-1α, and the results showed ALDH3A1 was positively correlated with HIF-1α (R = 0.39, *P* = 0.0056; Fig. 1H). Therefore, we concluded that highly expressed ALDH3A1 was induced by hypoxia and might regulate glycolysis in NSCLC.

### ALDH3A1 promotes cell proliferation and regulates energy metabolism reprogramming in NSCLC

**Fig. 2:**
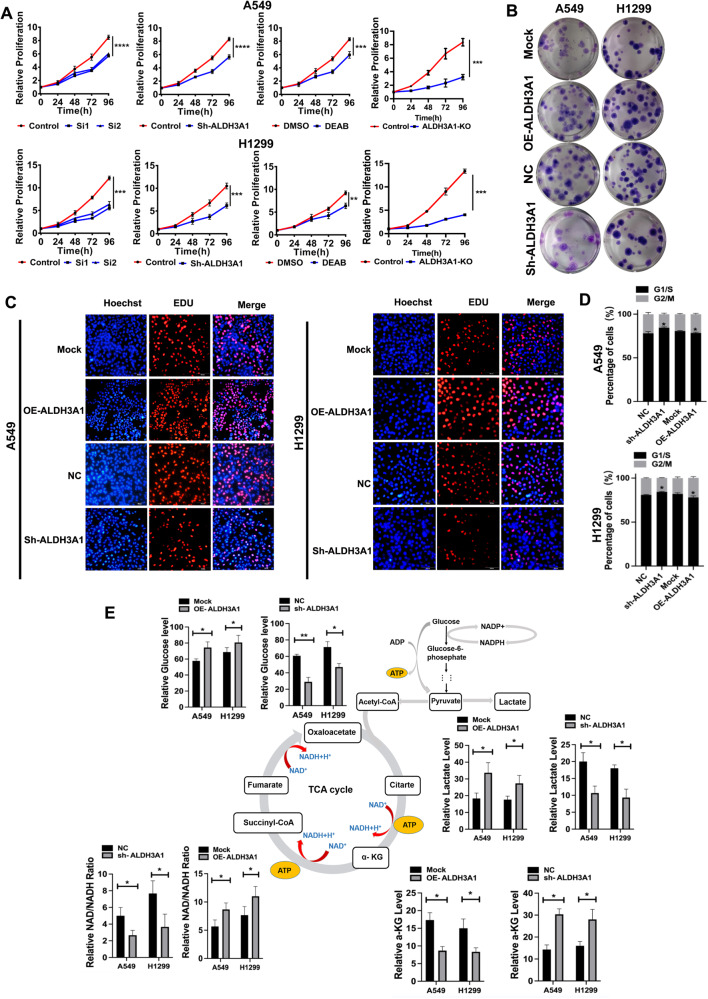
ALDH3A1 promotes cell proliferation and regulates the reprogramming of energy metabolism in NSCLC. **A** MTT assay showed the effects of ALDH3A1 on the proliferation of NSCLC. **B** Clone formation assay indicated the effects of ALDH3A1 on the proliferation of NSCLC. **C** EDU assay indicated the effects of ALDH3A1 on the proliferation of NSCLC. **D** Effect of ALDH3A1 on the cell cycle distribution of A549 and H1299 cells. Data are presented as mean ± SD (*n* = 3). Student’s *t*-tests were used for statistical analyses. **E** The respective effects of ALDH3A1 on the metabolites in glycolysis. *P < 0.05, **P < 0.01, ***P < 0.001.

To identify the functions of ALDH3A1 in NSCLC, si-ALDH3A1, stable ALDH3A1 knockdown, and ALDH3A1 knockout and overexpression cell lines were constructed (Supplementary Fig. S2A–E). The MTT assays demonstrated that the proliferation of NSCLC cells was reduced in the ALDH3A1 knockdown cells, and the effect was more significant for ALDH3A1-KO. Similar results were obtained by incubating cells with an ALDH3A1 inhibitor, DEAB (Fig. 2A). ALDH3A1 expression significantly promoted the growth of A549 and H1299 cells in our experiments using colony formation assays (Fig. 2B). The EDU assay showed that ALDH3A1 expression increased the replication of DNA in NSCLC (Fig. 2C). Flow cytometric analysis was carried out, and the results showed that ALDH3A1 expression activated the G1 phase in A549 and H1299 cells (Fig. 2D). Further, whereas bioinformatics suggests that ALDH3A1 is related to glycolysis, we found that ALDH3A1 expression could upregulate the level of glucose uptake, lactate level, and NAD^+^/NADH ratio, while it could downregulate the level of α-KG (Fig. 2E). The seahorse results showed that si-ALDH3A1 could significantly decrease ECAR and slightly increase OCR in both the A549 and H1299 cells (Supplementary Fig. S2F, G). The opposite results were obtained in the case of the ALDH3A1 knockdown cells (Fig. 2B–E).

### ALDH3A1 promotes cell proliferation via regulating energy metabolism reprogramming in NSCLC

**Fig. 3:**
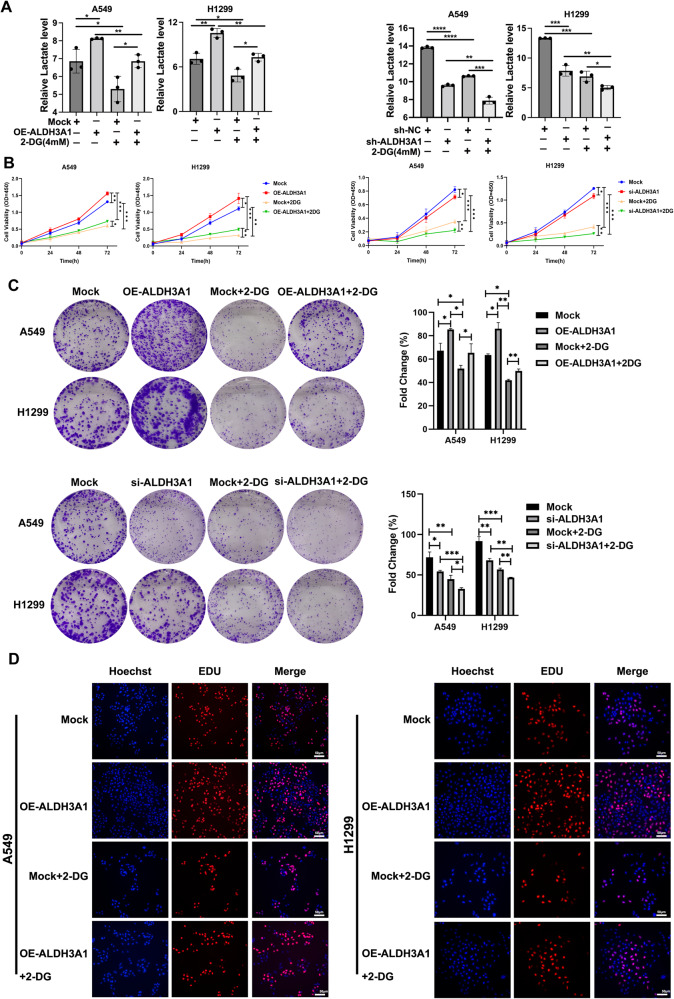
ALDH3A1 promotes cell proliferation via enhancing glycolysis in NSCLC. **A** Lactate production in A549 and H1299 cells in the presence of 2-DG. **B** CCK-8 showed the effects of 2-DG on the proliferation of A549 and H1299 cells. **C** Clone formation assay indicated the effects of 2-DG on the proliferation of A549 and H1299 cells. **D** EDU assay indicated the effects of 2-DG on the proliferation of A549 and H1299 cells. *P < 0.05, **P < 0.01, ***P < 0.001.

To investigate whether the reprogramming of energy metabolism was responsible for the progression of NSCLC, ALDH3A1 knockdown and overexpression cells were treated with 2-DG. The results showed that 2-DG significantly inhibited glycolysis in the ALDH3A1 knockdown and overexpression cells (Fig. 3A). Further, the CCK-8, colony formation, and EDU assay showed that compared with the ALDH3A1 overexpression group, the cell proliferation ability of the combination of ALDH3A1 overexpression and the 2-DG group was also decreased, and the opposite results were obtained for the ALDH3A1 knockdown cells (Fig. 3B–D). These results indicated that ALDH3A1 promotes cell proliferation by enhancing glycolysis in NSCLC cells.

### ALDH3A1 is a potential target of β-elemene in NSCLC

**Fig. 4:**
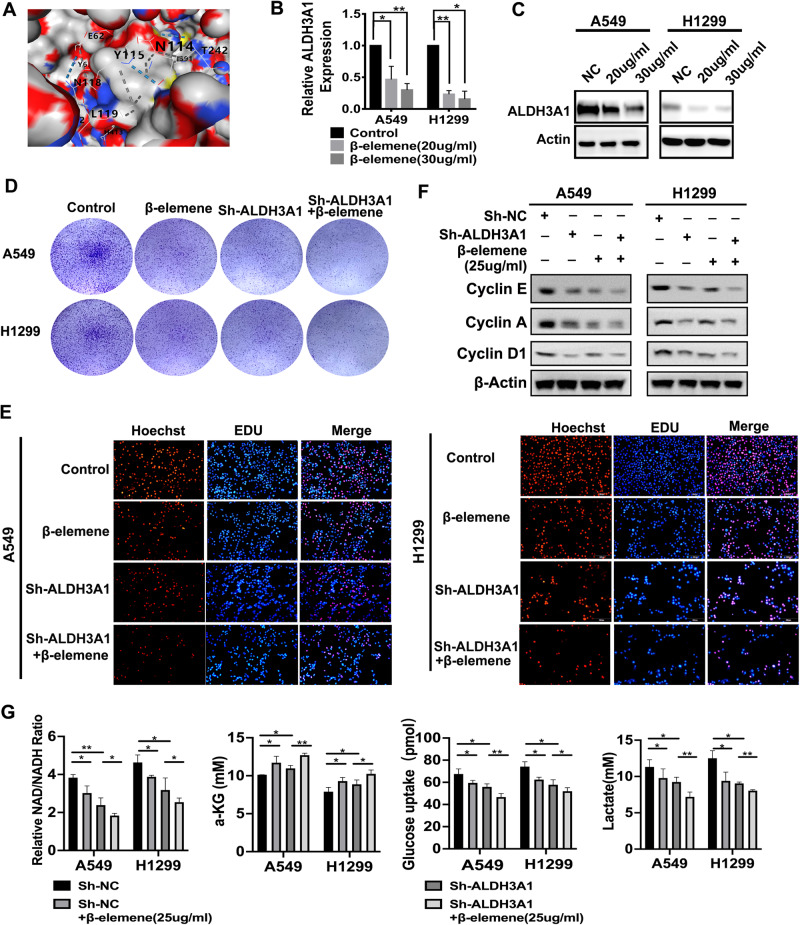
ALDH3A1 is a potential target of β-elemene. **A** Molecular docking of ALDH3A1 and β-elemene. **B**, **C** The expression of ALDH3A1 on the mRNA and protein levels after treatment with β-elemene. **D** The clones were enumerated when NSCLC cells were treated with sh-ALDH3A1 and β-elemene. **E** EdU assay was conducted using two cell lines with sh-ALDH3A1 and β-elemene treatment. **F** NSCLC cells transduced with sh-NC and sh-ALDH3A1 with or without β-elemene were subjected to Western blotting to identify the cell cycle proteins. **G** The respective effects of β-elemene, sh-ALDH3A1, and their combination on the metabolites in glycolysis. *P < 0.05, **P < 0.01, ***P < 0.001.

Our previous studies have shown that β-elemene can be used to inhibit tumor progression [16–19]. Therefore, according to the theory of network pharmacology analysis, we identified nine important targets of β-elemene in NSCLC via PPI network construction and KEGG pathway enrichment analysis (Supplementary Fig. S3A–C). Furthermore, through the expression of the targets in NSCLC, combined with the molecular docking results and previous studies (Supplementary Fig. S3D) [23], we finally determined that the core target of β-elemene in NSCLC might be ALDH3A1. The molecular docking results showed that ALDH3A1 could bind to β-elemene at five sites with a high binding affinity (Fig. 4A, Supplementary Table S2). Furthermore, β-elemene was used to stimulate A549 and H1299 cells, and the results indicated that ALDH3A1 expression was downregulated by β-elemene in a dose-dependent manner in NSCLC (Fig. 4B, C). On a deeper level, a rescue experiment showed that the combination of sh-ALDH3A1 and the β-elemene group caused a more potent inhibition of cell proliferation and DNA replication (Fig. 4D–F), a higher α-KG (α-ketoglutarate) level, and a lower NAD^+^/NADH ratio and lactate level than the other groups (Fig. 4E). From the above, we can see that ALDH3A1 expression can be downregulated by β-elemene to inhibit glycolysis and enhance OXPHOS, thus suppressing cell proliferation in NSCLC.

### ALDH3A1 reprograms energy metabolism via the HIF-1α/LDHA pathway

**Fig. 5:**
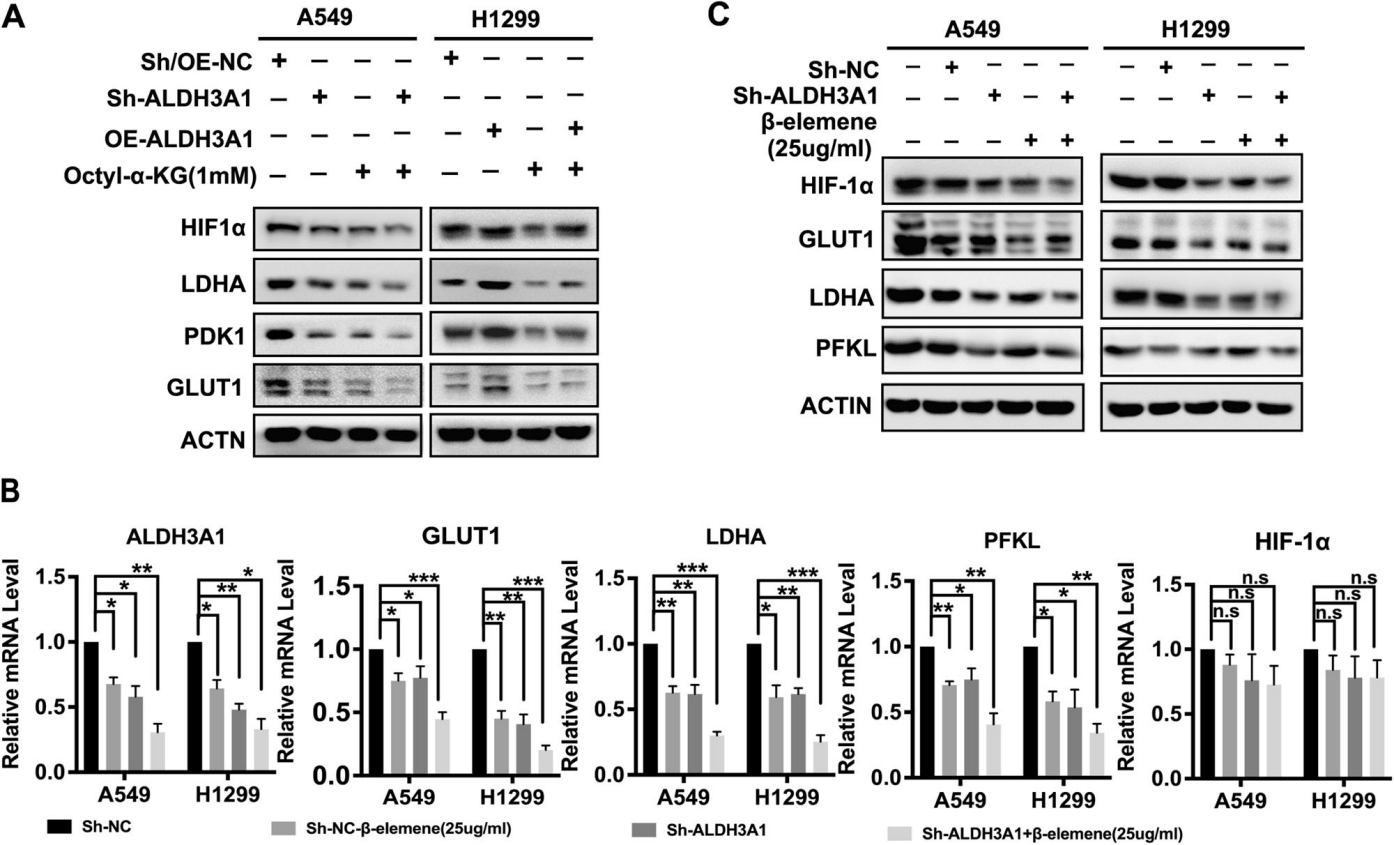
ALDH3A1 reprograms energy metabolism via the HIF-1α/LDHA pathway. **A** Western blotting was used to detect the protein expression associated with the HIF-1α/LDHA pathway of NSCLC cells transduced with Sh-NC and Sh-ALDH3A1, as well as OE-NC and OE-ALDH3A1, with and without treatment with Octyl-α-KG (1 mM). **B** Combinatorial treatment effects of β-elemene and Sh-ALDH3A1 on glycolysis genes on the mRNA level. **C** Western blotting was used to detect protein expression associated with the HIF-1α/LDHA pathway of NSCLC cells transduced with sh-NC and sh-ALDH3A1 with and without treatment with β-elemene (25 μg/ml). *P < 0.05, **P < 0.01, ***P < 0.001.

The HIF-1α/LDHA pathway is regarded as a critical signaling pathway in glycolysis [24, 25]. To explore its mechanism, ALDH3A1 was separately knocked down and overexpressed in A549 and H1299 cells based on its expression. The results indicated that the knockdown of ALDH3A1 could downregulate the expression of HIF-1α, LDHA, PDK1, and GLUT1, which could be strengthened via treatment with α-KG in the A549 cells, while the opposite results were observed in the H1299 cell (Fig. 5A). Further study demonstrated that a combination treatment of sh-ALDH3A1 and β-elemene significantly reduced the expression of GLUT1, LDHA, and PFKL, but not HIF-1α, in the mRNA and the protein level, rather than treating the groups alone (Fig. 5B, C). All these data suggest that ALDH3A1 promotes glycolysis and inhibits OXPHOS by activating the HIF-1α/LDHA pathway in NSCLC, which can be suppressed by β-elemene.

### ALDH3A1 regulates metabolic reprogramming and thus promotes cell proliferation through the HIF-1α/LDHA pathway in vivo

**Fig. 6:**
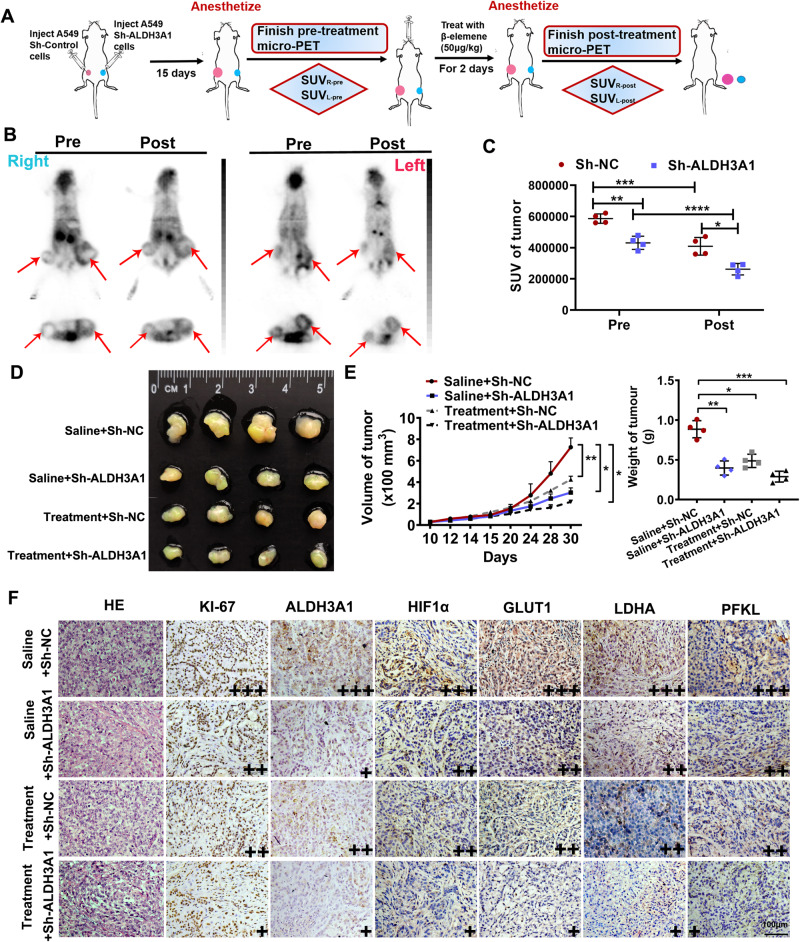
Experimental validation in vivo. **A** The flow diagram of the construction of the mice model to identify the efficacy of ALDH3A1. **B** The ^18^FDG micro-PET scan was used for the mice model treated with β-elemene and/or sh-ALDH3A1. **C** The SUV values of the tumors in vivo. **D** Photographs of tumors after the injection of Sh-NC (left) and Sh-ALDH3A1 (right) with and without β-elemene treatment. **E** The weight and volume of the tumors. **F** Xenograft tumor tissues treated for 15 days, as indicated, were evaluated via hematoxylin and eosin staining and immunohistochemistry (×200). *P < 0.05, **P < 0.01, ***P < 0.001.

To verify the results in vitro, a xenograft model was constructed (Fig. 6A). An ^18^FDG micro-PET scan was used to validate the metabolic level 2 days after treatment with β-elemene. Compared with sh-control, treatment with sh-ALDH3A1 or β-elemene alone resulted in reductions in the SUV values of the tumors. Furthermore, the combination of sh-ALDH3A1 and β-elemene markedly suppressed glucose uptake in the tumors, whose SUV values decreased sharply (Fig. 6B, C). The mice treated with β-elemene every other day were sacrificed after 2 weeks. Consistently, the downregulation of ALDH3A1 or treatment with β-elemene alone could decrease the volumes and weights of the tumors. Moreover, the combination of sh-ALDH3A1 and β-elemene caused a more dramatic inhibition of tumor growth (Fig. 6D, E). The tumor tissues were then subjected to immunohistochemical staining to detect proteins associated with the cell cycle, Ki-67, and the HIF-1α/LDHA pathway (Fig. 6F). These results were consistent with those obtained in vitro. Specifically, ALDH3A1 enhances glycolysis to promote the cell proliferation of NSCLC by activating the HIF-1α/LDHA pathway, whereas this effect could be suppressed by β-elemene in vivo.

## Discussion

The metabolic reprogramming of aerobic glycolysis leads to a growth advantage for cancer cells by providing energy for their growth. Therefore, the identification of carcinogenic signals responsible for the reprogramming of glucose metabolism may be translated into better antitumor therapies. In this study, the results supported the notion that hypoxia-induced ALDH3A1 reprogrammed glycolysis and OXPHOS balance to promote cell proliferation through the HIF-1α/LDHA pathway in NSCLC. In addition, our study proved that ALDH3A1 is a target of β-elemene, which is a promising drug for the treatment of NSCLC.

Previous studies have demonstrated that the expression of ALDH3A1 was higher in lung tumor tissues, which is related to a poor prognosis [26], a finding consistent with our study.

ALDHs play an important role in the detoxification of alcohol-derived acetaldehyde. They are involved in the metabolism of corticosteroids, biogenic amines, neurotransmitters, and lipid peroxidation. They are strongly associated with poor prognosis in alcohol-related tumors, such as esophageal squamous cancer [27] and oral squamous cancer [9]. However, some studies have reported ALDH3A1 as one of the stem cell markers that is strongly associated with a poor prognosis in tumors [10]. Given that its TCGA data in lung adenocarcinoma only showed a trend of high expression in tumors, we searched the GSE18842 dataset further and collected samples of lung adenocarcinoma for validation, and we found that the high expression of ALDH3A1 in lung adenocarcinoma was also associated with a poor prognosis.

Hypoxia is a hallmark of metastasis-promoting solid tumors and is a significant barrier to successful cancer treatment. During our preliminary literature research, we found that cancer cells activate transcriptional programs in response to hypoxia, enabling them to survive in this harsh microenvironment. HIF-1α is thought to be the primary effector of the cellular response to hypoxia, stimulating the transcription of genes involved in promoting angiogenesis and altering cellular metabolism. However, a growing body of evidence further suggests that the cellular response to hypoxia is much more complex, involving coordinated signaling through stress-responsive pathways. A key signaling molecule activated in response to hypoxia is the nuclear factor erythrocyte 2-like-2 (NRF2) [28, 29]. HIF-1α and NRF2 have distinct and overlapping roles in the cellular response to hypoxia, and emerging evidence suggests that coordinated signaling through NRF2 and HIF-1α is critical for tumor survival and progression and that the ALDH family is one of the coordinating signals. In recent years, it has been shown that NRF2 acts as a transcription factor for ALDH3A1 and induces NADH reduction stress by upregulating the NAD^+^-depleting enzyme ALDH3A1 [30]. This is an important insight for our future research.

This research suggested that a hypoxic environment could induce gene differential expression to affect cell glycolysis, thus regulating tumor progression [31–33]. We also found that the expression of ALDH3A1 was significantly upregulated in a hypoxia time-dependent manner. ALDH3A1 is known as an important marker for cancer stem cells [34, 35]. In addition, ALDH3A1 is closely associated with elevated cellular metabolism as a NAD(P)^+^-dependent enzyme [36]. A recent study reported that ALDH3A1 promoted the metastasis of lung cancer by accelerating the glycolytic process [13]. The results of our GSEA enrichment analysis verified that ALDH3A1 was positively correlated with the process of the cell cycle and glycolysis. Furthermore, we observed that ALDH3A1 facilitated the proliferation of NSCLC by regulating cell metabolism in vitro and in vivo. Specifically, we identified that the knockdown of ALDH3A1 could downregulate the level of glucose uptake, lactate level, and NAD^+^/NADH ratio while it upregulated the level of α-KG. In addition, the knockdown of ALDH3A1 could downregulate the expression of HIF-1α, LDHA, PDK1, and GLUT1, which could be further strengthened by treatment with α-KG. It has been reported that, as a key enzyme in the dynamic balance of NAD^+^ and NADH, ALDH3A1 first changes the ratio of NAD^+^ and NADH and then blocks the tricarboxylic acid cycle (TAC), which decreases the level of α-KG, leading to the upregulation of HIF-1α and its downstream pathway [37, 38], a finding which is consistent with our conclusion and provides strong evidence for our results. Generally speaking, our study demonstrates that ALDH3A1 promotes glycolysis and inhibits OXPHOS to facilitate cell proliferation via activation of the HIF-1α/LDHA pathway in NSCLC.

Many of our group’s studies on the clinical applications of β-elemicene have been published. β-Elemicene can inhibit the peritoneal metastasis of gastric cancer by inhibiting FAK activation, as well as reverse multidrug chemoresistance by modulating the miR-1323/Cbl-b/EGFR pathway, in addition to sensitizing chemotherapeutic agents [39, 40]. A recent study indicated that β-elemene attenuates the Warburg effect in NSCLC cells, possibly by mediating the miR-301a-3p/AMPKα axis [41]. Similarly, the results of our in vivo and in vitro experiments support the notion that β-elemene reduced NSCLC proliferation by reprogramming the balance of glycolysis and OXPHOS. Moreover, ALDH3A1 was identified as a target of β-elemene in vitro and in vivo. Thus, patients with high ALDH3A1 expression may be a population that can benefit from β-elemene, and our study also suggests that PET/CT may be one of the effective noninvasive testing tools for the initial screening of this group of patients. Of course, this conclusion requires further confirmation based on relevant clinical studies.

In conclusion, we found that hypoxia could induce the expression of ALDH3A1 in NSCLC for the first time. It was first demonstrated that ALDH3A1 enhanced glycolysis and inhibited OXPHOS to promote cell proliferation by activating the HIF-1α pathway both in vivo and in vitro. ALDH3A1 was identified as a target of β-elemene. This study provides important clues for the study of the pathogenesis of NSCLC and lays a solid foundation for the search for precision treatment targets and effective therapeutic drugs for this disease.

### Supplementary information

Supplementary materials

aj-checklist

Original Data File

## Data Availability

The original contributions presented in the study are included in the article and Supplementary Material. Further inquiries can be directed to the corresponding authors.
