# Supplementary material for: Hypoxia-induced ALDH3A1 promotes the proliferation of non-small-cell lung cancer by regulating energy metabolism reprogramming
Source: Cell Death Dis. 2023 Sep 20;14(9):617. doi: 10.1038/s41419-023-06142-y (PMC10511739; doi:10.1038/s41419-023-06142-y)
Supplement: Supplementary file 1 — Supplementary materials [file 41419_2023_6142_MOESM1_ESM.docx]

**Supplementary materials**

**Hypoxia-induced ALDH3A1 promotes proliferation of non-small cell lung cancer by regulating energy metabolism reprogramming**

| **Table S1. The primer sequence used for qRT-PCR.** | |
| --- | --- |
| **Primer name** | **Sequence** |
| ALDH3A1 |  |
| Forward | TGTTCTCCAGCAACGACAAGG |
| Reverse | GTGGGAATGTGAGCTGGAAC |
| HIF-1α |  |
| Forward | TGTTCTCCAGCAACGACAAGG |
| Reverse | GTGGGAATGTGAGCTGGAAC |
| GLUT1 |  |
| Forward | TGTTCTCCAGCAACGACAAGG |
| Reverse | GTGGGAATGTGAGCTGGAAC |
| PFKL |  |
| Forward | TGTTCTCCAGCAACGACAAGG |
| Reverse | GTGGGAATGTGAGCTGGAAC |
| LDHA |  |
| Forward | TGTTCTCCAGCAACGACAAGG |
| Reverse | GTGGGAATGTGAGCTGGAAC |
| 18S |  |
| Forward | AGGGCAGAGAGTGCAAGGT |
| Reverse | CGCCCGCCCGCTCCCAAGAT |
| qRT-PCR, quantitative real-time polymerase chain reaction. | |

| **Table S2. The molecular docking of β-elemene and ALDH3A1.** | | |
| --- | --- | --- |
| **Site** | **Vina score** | **Cavity size** |
| 1 | -6.6 | 8762 |
| 2 | -6.4 | 10586 |
| 3 | -6.2 | 10327 |
| 4 | -6.1 | 10150 |
| 5 | -5.9 | 1473 |

**
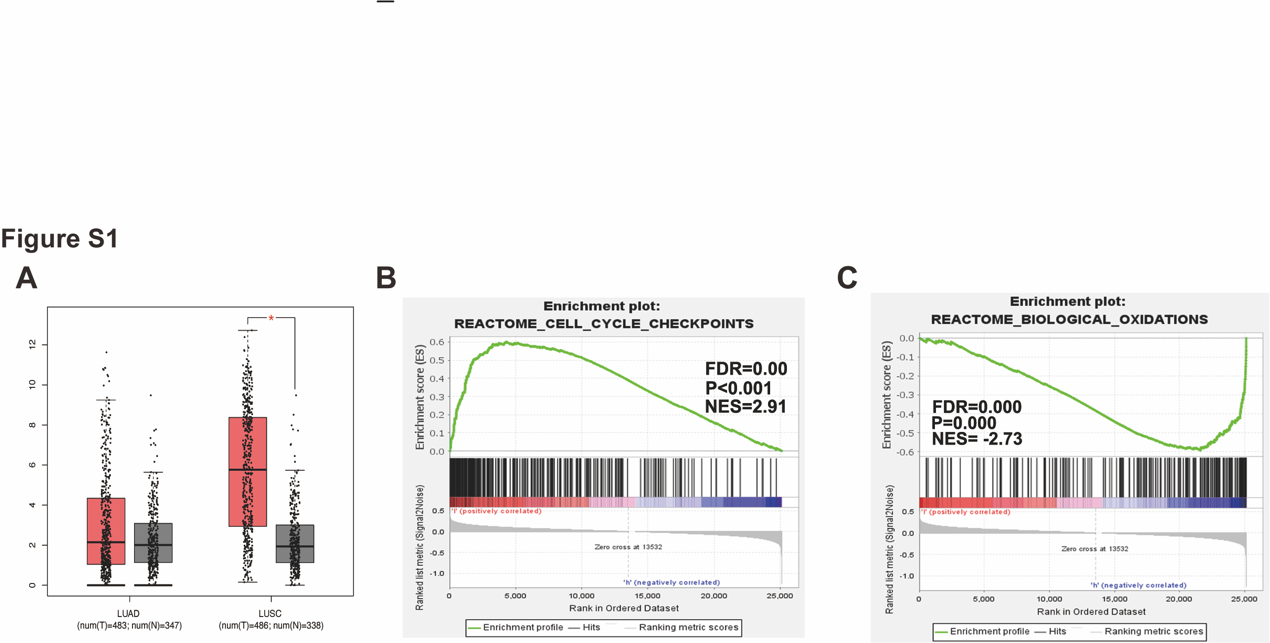
**

**Figure.S1.** **Expression and molecular function of ALDH3A1.** A. ALDH3A1 expression in normal tissues and NSCLC. B-C. GSEA enrichment analysis of ALDH3A1 in NSCLC with TCGA dataset.

**
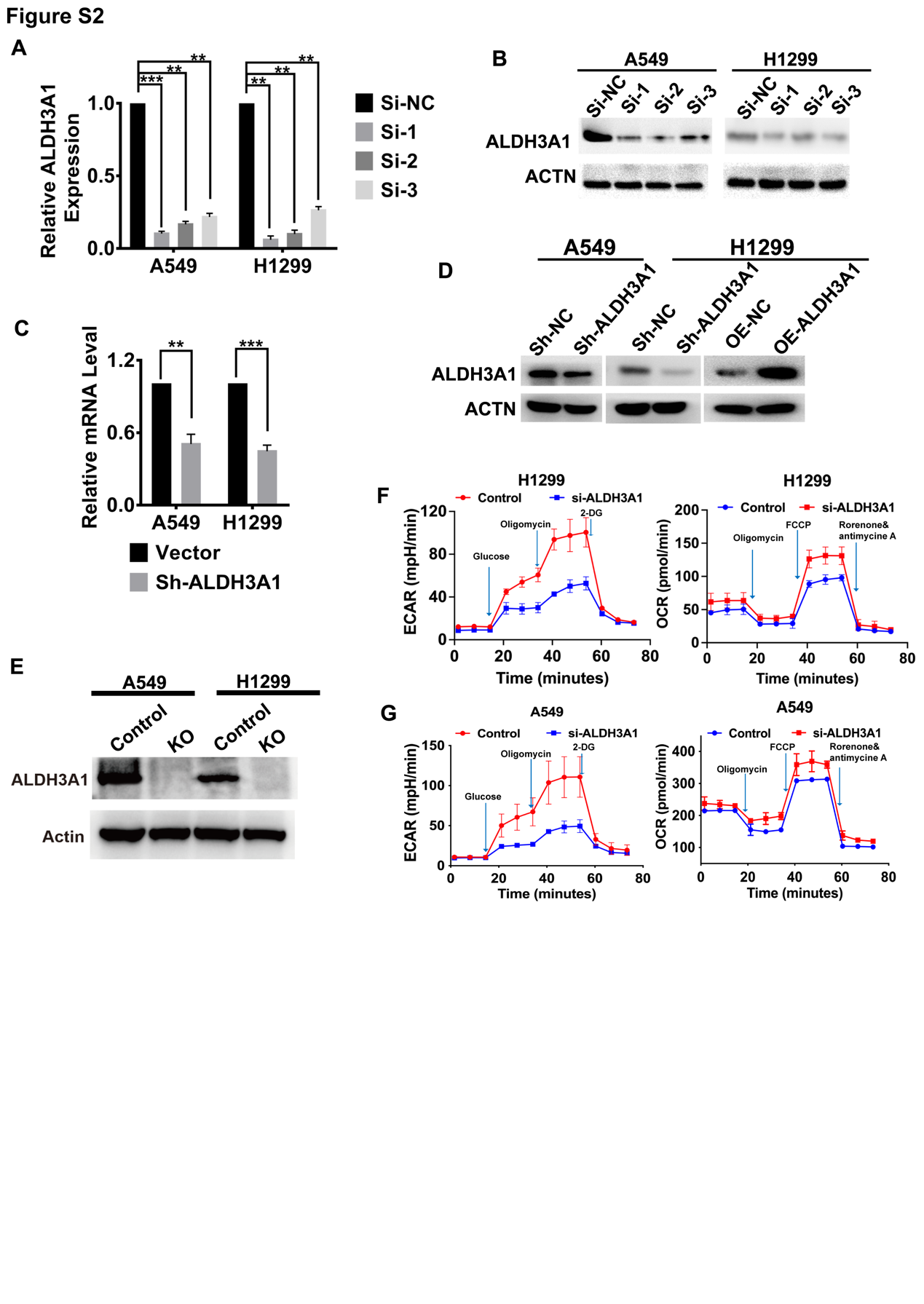
**

**Figure.S2：**A-B. The knockdown rate of si-ALDH3A1 at the RNA and protein levels. C-D. The knockdown rate of Sh-ALDH3A1 at the RNA and protein levels and the overexpression rete of ALDH3A1 at protein levels. E. The knockout rate of ALDH3A1-KO at protein level.


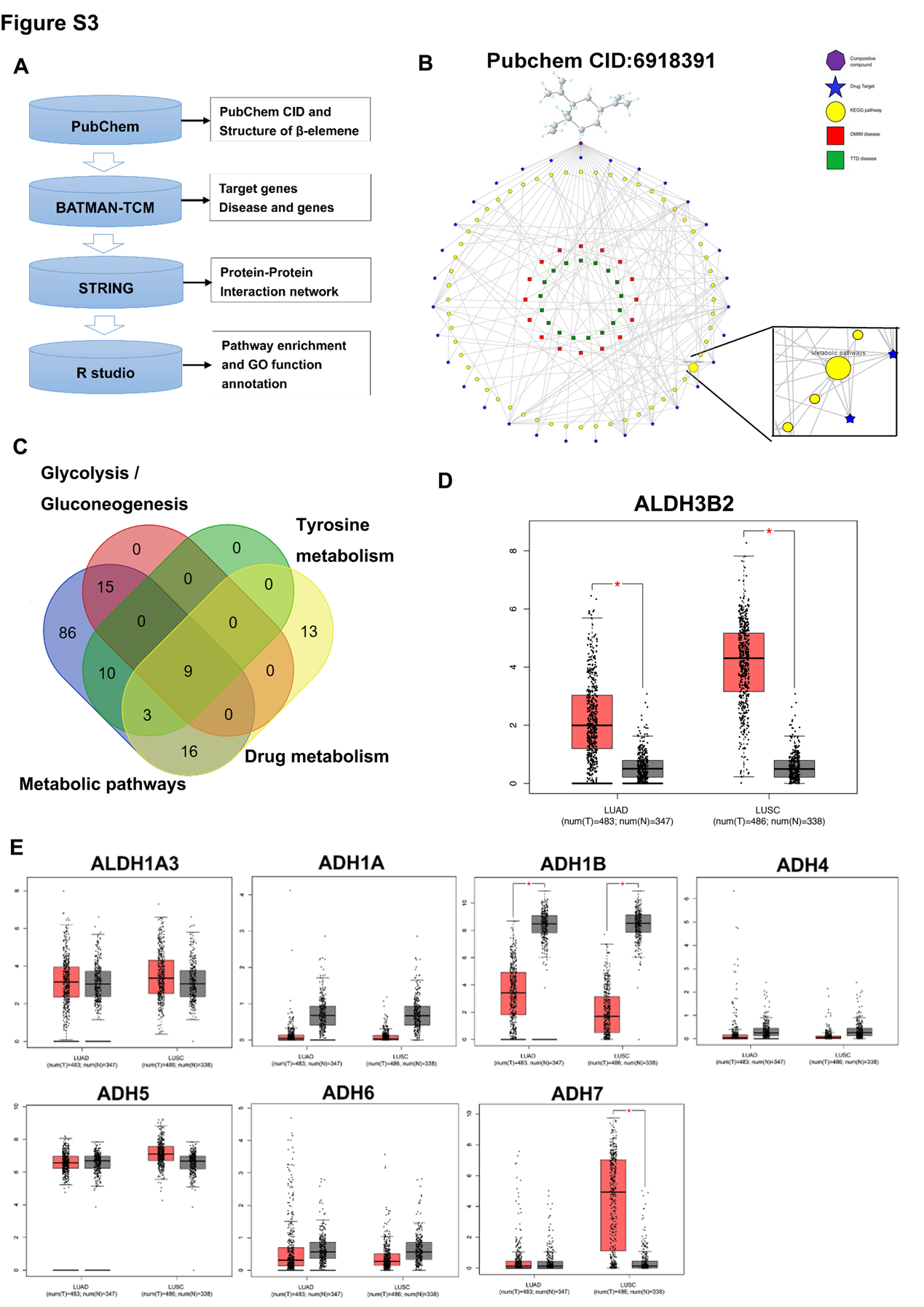


**Figure S3.** A. Flowchart for screening of β-elemene core targets. B. Bioinformatics Analysis Tool for Molecular Mechanism of Traditional Chinese Medicine (BATMAN-TCM). C. Venn diagram of the top 4 KEGG pathways that were affected by β-elemene. D. ALDH3B2 expression in normal tissues and NSCLC. E. Other 7 predicted targets expression in NSCLC. *P<0.05, **P<0.01,***P<0.001.
