# Supplementary figures and images for: Hypoxia-induced ALDH3A1 promotes the proliferation of non-small-cell lung cancer by regulating energy metabolism reprogramming
Source: Cell Death Dis. 2023 Sep 20;14(9):617. doi: 10.1038/s41419-023-06142-y (PMC10511739; doi:10.1038/s41419-023-06142-y)

Figure1:


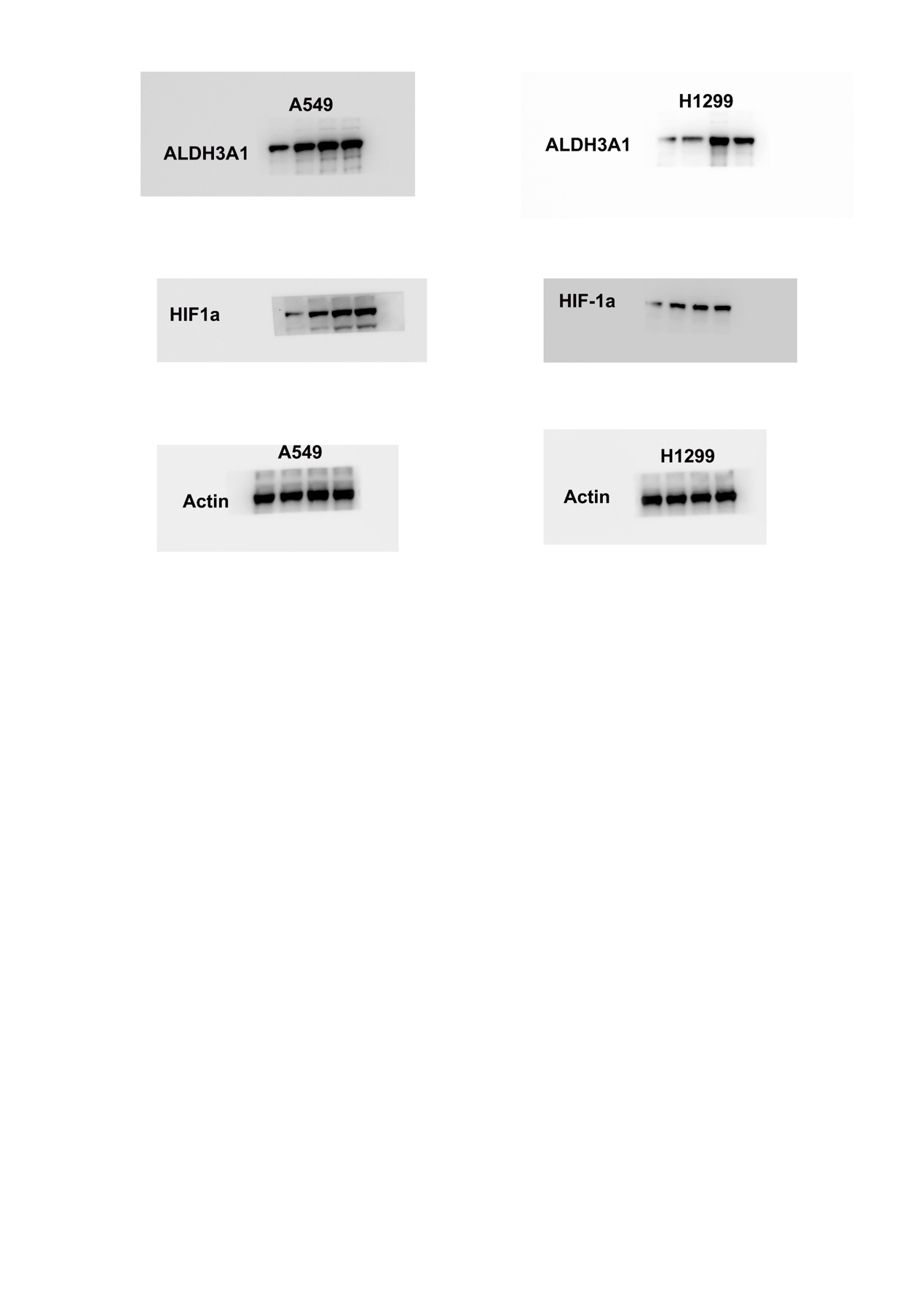


Figure4:


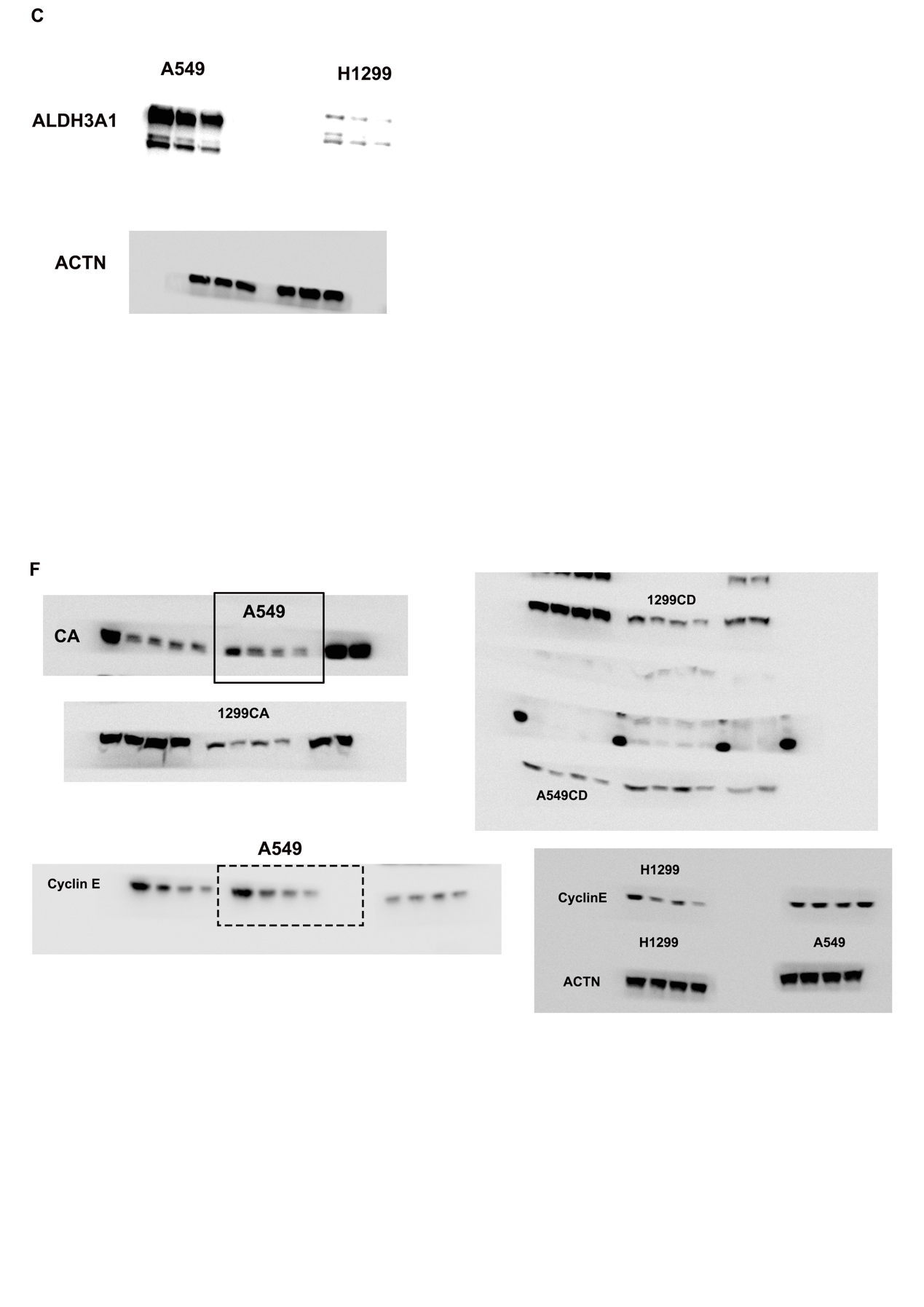


FigureS2


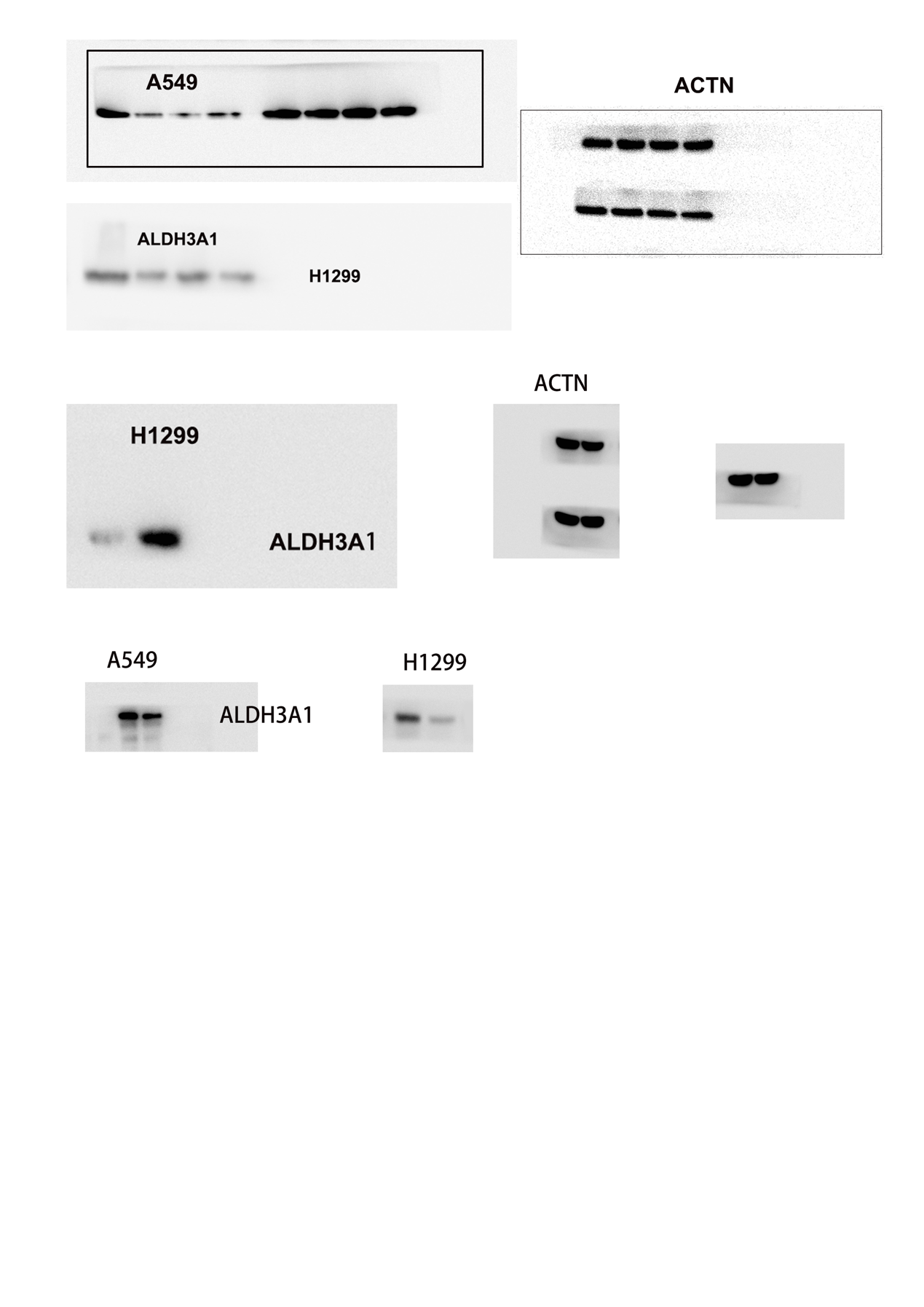


FigureS3


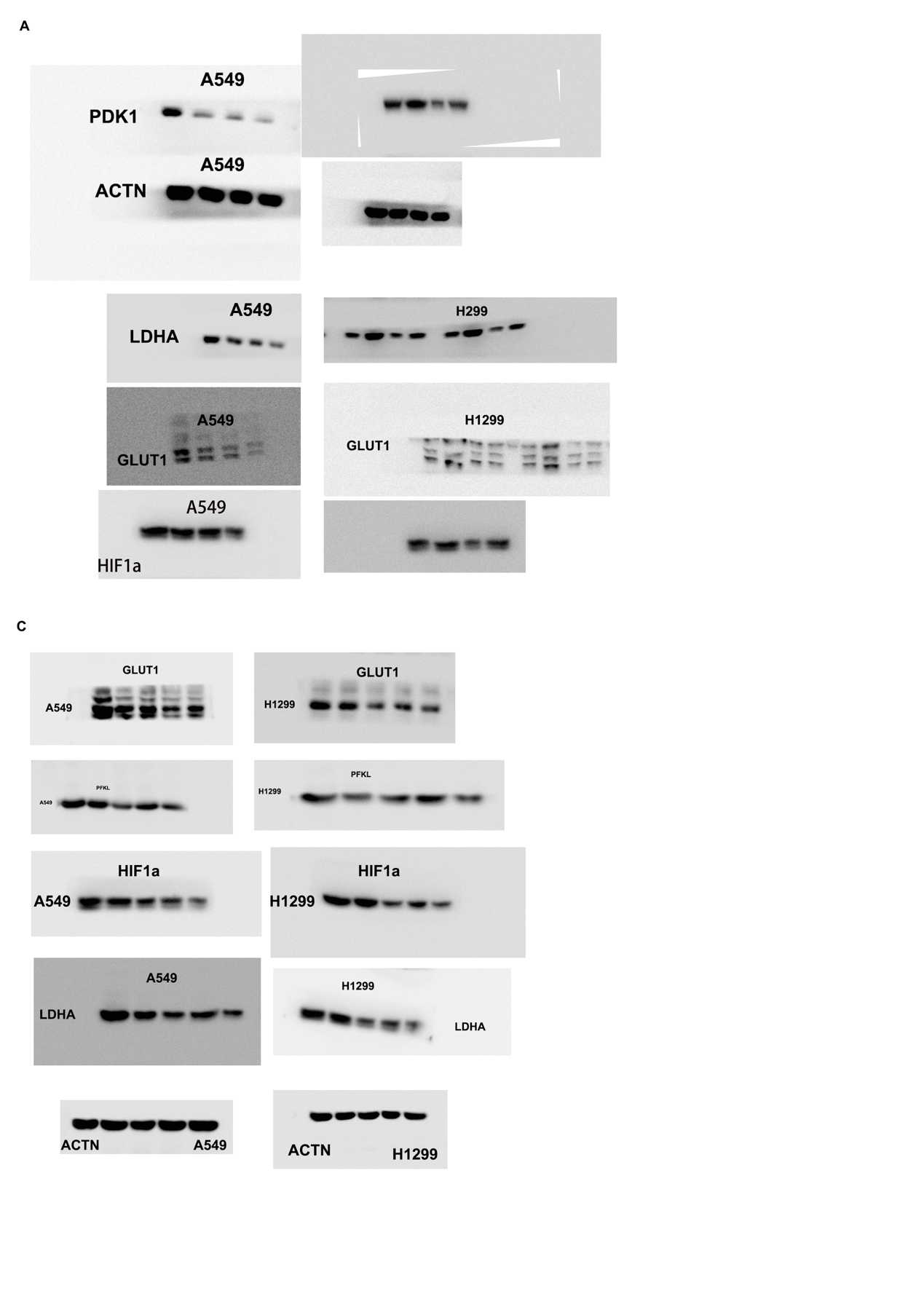

Supplement: Supplementary file 3 — Original Data File [file 41419_2023_6142_MOESM3_ESM.docx]
